# Treating Infected Non-Healing Venous Leg Ulcers with Medical-Grade Honey: A Prospective Case Series

**DOI:** 10.3390/antibiotics13070614

**Published:** 2024-07-02

**Authors:** Georgios E. Papanikolaou, Georgios Gousios, Niels A. J. Cremers, Linsey J. F. Peters

**Affiliations:** 1 GP Plastic Surgery Private Practice, 45444 Ioannina, Greece; 2 PharmaLife, 45444 Ioannina, Greece; g.gousios100@gmail.com; 3 Department of Gynecology and Obstetrics, Maastricht University Medical Centre, P. Debyelaan 25, 6229HX Maastricht, The Netherlands; 4 Triticum Exploitatie BV, Sleperweg 44, 6222NK Maastricht, The Netherlands; research@mesitran.com

**Keywords:** antimicrobials, bacterial colonization, chronic wounds, elderly patients, venous insufficiency, wound infection

## Abstract

Venous leg ulcers (VLUs) are hard-to-heal wounds and are prone to microbial colonization. Innovative and improved therapies are thus required to resolve local infection and enhance the wound healing process. The objective of this study was to evaluate the effectiveness of medical-grade honey (MGH) for the treatment of clinically infected and non-healing VLUs. This prospective case series included nine patients with an average age of 83.4 years (range: 75–91 years) with a total of eleven VLUs, previously ineffectively treated with various products. Major risk factors for the appearance of VLUs were chronic venous insufficiency, advanced age, multiple comorbidities (particularly cardiovascular diseases), and impaired mobility. All wounds presented with local signs of infection. Upon presentation, treatment was commenced with a range of MGH-based products (L-Mesitran^®^). Clinical signs of infection were eliminated by MGH after 2.2 weeks on average (range: 1–4 weeks), and wounds were completely healed after 7 weeks on average (range: 3–18 weeks). No further complications or recurrences were observed. MGH has a broad-spectrum antimicrobial activity and promotes rapid healing, thus improving patients’ quality of life. Moreover, MGH-based products are safe, easy to use, cost-effective, and can effectively treat VLUs alone or in combination with standard-of-care therapies.

## 1. Introduction

Venous leg ulcers (VLUs) are defined as a partial or full-thickness defect of the skin and/or the underlying soft tissues located below the knee and typically on the gaiter region of the lower leg. These patients usually present with chronic venous insufficiency (CVI) associated with valvular incompetence and failure of the calf muscle pump, thus resulting in vein dilatation and venous hypertension [1]. The resultant microangiopathy causes the accumulation of leukocytes and the release of inflammatory mediators, leading to local tissue injury characterized by dermal hyperpigmentation, sclerosis, and skin ulceration [2].

The prevalence of VLUs is up to 1.69%, and the incidence is up to 1.33% of the population, while they are the most common ulcer of the lower extremity, accounting for between 70 and 80% of all leg ulcers [2,3]. Chronicity of VLUs constitutes a major global health issue since these hard-to-heal wounds can persist for at least 4 weeks, without a tendency to heal despite the use of appropriate treatments. Currently, at least 60% of VLUs result in a chronic wound, while the healing rates can be protracted, with fewer than 60% healed by 12 weeks [4,5]. Elderly patients with severe comorbidities are more prone to microbial contamination and therefore biofilm formation resulting in protracted periods of infected and non-healing lower leg wounds [6]. However, once healed, VLUs present a high recurrence rate, which can be as high as 50–70% at 6 months [7].

Patients with VLUs experience low quality of life, they must face an economic loss associated with increased missed workdays, and they utilize more medical resources, increasing the public health care cost [8,9]. Therefore, a holistic approach must be applied either to prevent or to effectively treat VLUs. Primarily, all those risk factors involved in the pathogenesis of the VLUs such as chronic venous insufficiency (CVI), deep vein thrombosis (DVT), obesity, and impaired mobility must be faced [10]. Consequently, patients require the appropriate treatment method to achieve complete wound healing within a short time.

Honey has been used for centuries to treat different disorders, particularly skin wounds [11]. Over the past decades, the therapeutical potential of honey has attracted the interest of numerous studies, resulting in the production of medical-grade honey (MGH), which is clean of pollutants, follows specific physicochemical characteristics, and is gamma-sterilized to meet the criteria for clinical usage [12]. MGH exerts broad-spectrum antimicrobial and strong antibiofilm properties, reducing local inflammation and bacterial burden [13,14,15]. Moreover, MGH has a strong ability to promote wound healing by enhancement of angiogenesis, formation of healthy granulation tissue, and re-epithelialization [16,17,18]. Although MGH has been used in various indications, the documentation about its application in complex geriatric patients with clinically infected VLUs is limited. Moreover, MGH is not common practice to treat this specific indication in many clinics and is often reserved as a last resort. To demonstrate the application and potency of MGH in complex geriatric patients with clinically infected VLUs, we present the current case series.

In this prospective case series, we evaluate the clinical efficacy and safety of MGH (L-Mesitran, Triticum Exploitatie BV, the Netherlands) for treating hard-to-heal clinically infected VLUs in elderly patients with multiple comorbidities. In all patients, previous treatments had failed, which can therefore be considered as the controls. Furthermore, we aimed to highlight the use of MGH as a monotherapy to promote rapid wound healing in patients with VLUs.

## 2. Results

### 2.1. Case 1

**Figure 1:**
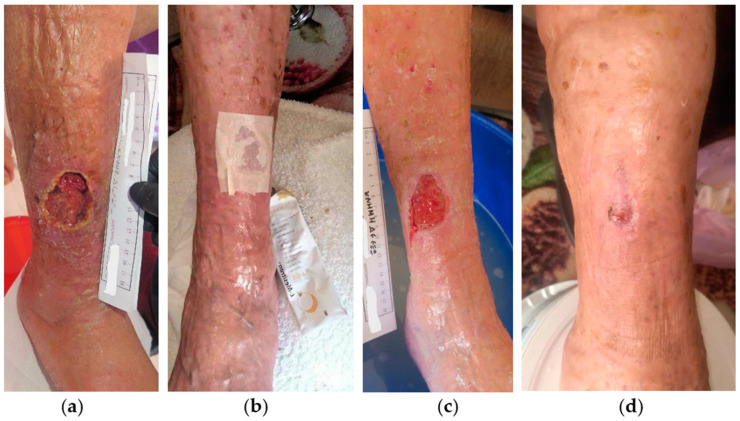
Case 1: (**a**) clinical findings at the initial examination, day 0 (start of MGH treatment); (**b**) wound dressing with L-MS, followed by L-MT; (**c**) follow-up examination after 4 weeks, with appearance of healthy granulation tissue, epithelialization, and elimination of local signs of infection; (**d**) complete wound healing after 11 weeks of MGH therapy.

A 79-year-old female patient presented with a stage III VLU at the outer surface of her right leg (Figure 1a). The lesion was aggravated due to an impact injury and consequently the formation of subcutaneous hematoma. The patient presented with limited mobility and several comorbidities, including CVI, arterial hypertension (AHT), heart failure, atrial fibrillation, hyperlipidemia, hyperuricemia, depression, chronic obstructive pulmonary disease (COPD), and vitamin D deficiency, while she was under anticoagulant (rivaroxaban 15 mg) and glucocorticoid (methylprednisolone 16 mg) therapy. The VLU was unsuccessfully treated with a povidone-iodine solution and mupirocin calcium cream for 4 weeks. Initially, the wound dimensions were 6 cm in length and 6 cm in width. Clinical signs of local infection included slough, high amount of exudate, unpleasant odor, delayed healing, pain, and erythema. Conservative treatment was initiated with L-Mesitran^®^ Soft wound gel (L-MS), followed by L-Mesitran^®^ Tulle (L-MT) to ensure contact with the wound bed (Figure 1b). A secondary foam dressing was applied to adequately address the high quantity of exudate. Wound dressings were performed by the healthcare professional at the patient’s home at 48 h intervals for the first three weeks. After 4 weeks, healthy granulation tissue and epithelialization were evident, and local signs of infection disappeared (Figure 1c). Consequently, the dressing changes were extended to every four days and were performed by the patient’s relatives at home. The VLU was completely healed after 11 weeks of MGH treatment without complications (Figure 1d).

### 2.2. Case 2

**Figure 2:**
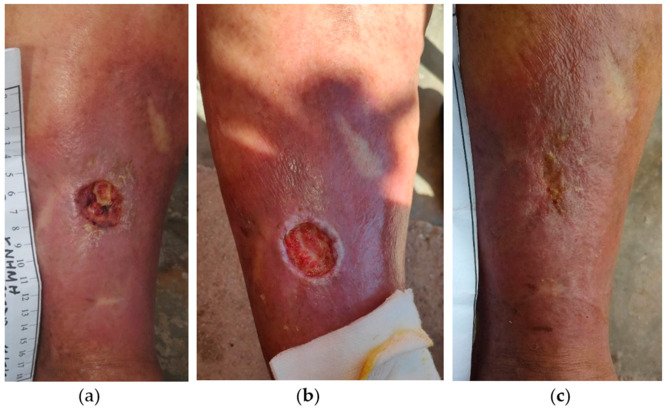
Case 2: (**a**) clinical findings at the initial examination, day 0 (start of MGH treatment); (**b**) the wound bed was clear and healthy granulation tissue was evident after 3 weeks of MGH treatment; (**c**) complete wound healing after 18 weeks of MGH therapy.

An 80-year-old female patient presented with a stage III VLU at the anterior surface of her right leg (Figure 2a). The patient’s medical history included CVI, AHT, osteoporosis, and obesity, and she was under anticoagulant therapy with acetylsalicylic acid 80 mg. Previously, the wound was treated with a povidone-iodine solution for about 20 weeks without any progress in the wound healing. On the initial presentation, the wound dimensions were 3 cm in length and 3 cm in width, with slough, moderate amount of exudate, peripheral skin discoloration, and edema. Local treatment was started with L-MS, followed by L-MT and a secondary foam dressing. Wound dressing changes were performed by the healthcare professional at the patient’s home at 48 h intervals for the first 2 weeks. Already after 3 weeks, the wound bed was cleared and started to fill with granulation tissue (Figure 2b). Consequently, dressing change intervals were extended to every 4 days. During the following weeks, the healing process was uneventful, and the VLU was completely healed after 18 weeks of MGH treatment (Figure 2c).

### 2.3. Case 3

**Figure 3:**
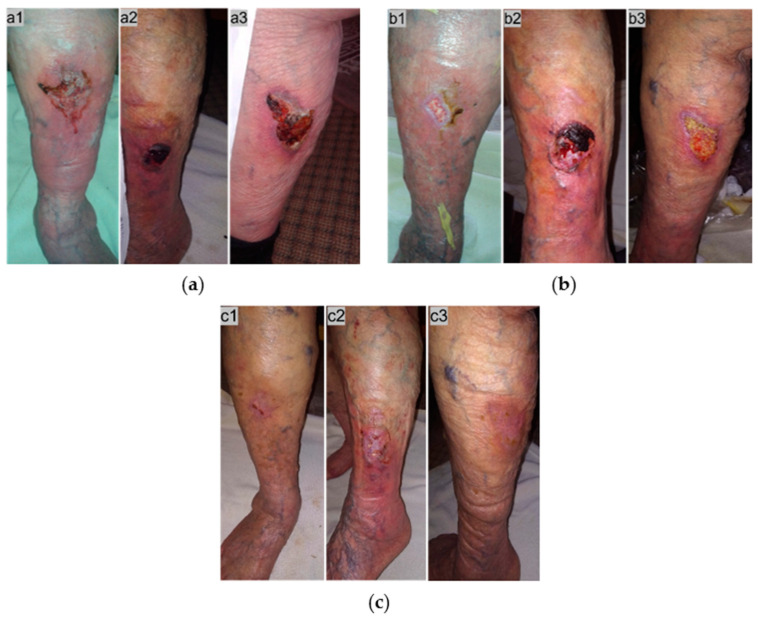
Case 3: (**a**) clinical findings at the initial examination, day 0 (start of MGH treatment), (**a1**) anterior surface of the right leg, (**a2**) lateral surface of the left leg, (**a3**) posterior surface of the left leg; (**b**) elimination of local signs of inflammation and appearance of healthy granulation tissue within 3 weeks of MGH therapy, (**b1**) anterior surface of the right leg, (**b2**) lateral surface of the left leg, (**b3**) posterior surface of the left leg; (**c**) complete wound healing within 6 weeks of MGH therapy, (**c1**) anterior surface of the right leg, (**c2**) lateral surface of the left leg, (**c3**) posterior surface of the left leg.

A 91-year-old female patient presented with multiple bilateral stage II VLUs along with varicose veins and local inflammation (Figure 3a). Relevant comorbidities included CVI, DVT, heart failure, atrial fibrillation, hyperlipidemia, hyperuricemia, asthma, and osteoporosis. The patient presented limited mobility and received anticoagulant (apixaban 2.5 mg, fondaparinux sodium 2.5 mg/0.5 mL) and glucocorticoid therapy (prednisolone 5 mg). On examination, the wounds had superficial necrotic tissue with indented edges, a moderate amount of exudate, peripheric edema, erythema, and pain. The VLUs were unsuccessfully treated for about 2 weeks with povidone-iodine solution. Dressings with L-MS in direct contact with the ulcer, followed by L-MT, and a secondary foam dressing, were initiated to resolve the local inflammation and promote healing. Wound dressing changes were performed by the healthcare professional twice a week. Within 3 weeks after MGH therapy started, all VLUs showed significant healing improvement (Figure 3b), with complete healing within 6 weeks (Figure 3c).

### 2.4. Case 4

**Figure 4:**
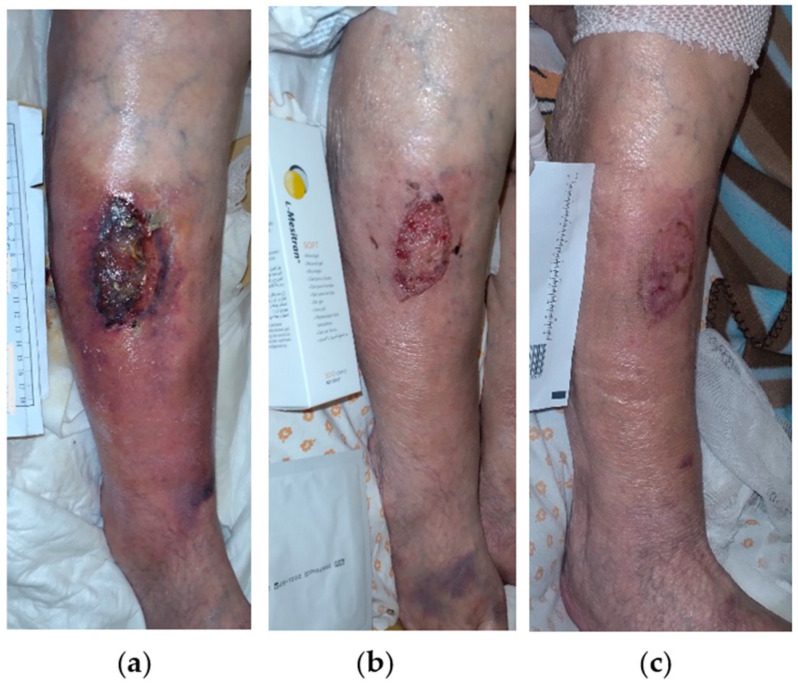
Case 4: (**a**) clinical findings at the initial examination, day 0 (start of MGH treatment); (**b**) resolution of the local infection, and advanced wound healing after 2 weeks of MGH therapy; (**c**) complete wound healing after 4 weeks of MGH therapy.

An 84-year-old female patient presented with a stage II VLU at the anterior surface of her right leg (Figure 4a). Medical comorbidities included chronic CVI, COPD, cerebrovascular disease (CVD), heart failure, atrial fibrillation, hyperuricemia, obesity, osteoarthritis, and osteoporosis. Moreover, the patient suffered from impaired ambulation and received anticoagulant therapy (acenocoumarol 4 mg). The VLU was in situ for about 4 weeks, without any local treatment. On initial observation, the wound dimensions were 9 cm in length and 6 cm in width, and it presented with signs of local infection including superficial necrosis, a moderate amount of exudate, and malodor, associated with peripheric signs of inflammation including erythema, hyperthermia, edema, and pain. Given the antimicrobial properties of MGH, treatment with L-MS, followed by L-MT and a foam dressing, was commenced, and dressing changes were performed at the patient’s home by the healthcare professional twice a week. During the next 2 weeks, local signs of infection gradually disappeared, peripheric inflammation resolved, and the wound area was replaced by granulation and epithelial tissue (Figure 4b). Due to the positive therapeutic response, dressing changes were continued as per the above protocol. After 4 weeks, the ulcer was completely healed uneventfully (Figure 4c).

### 2.5. Case 5

**Figure 5:**
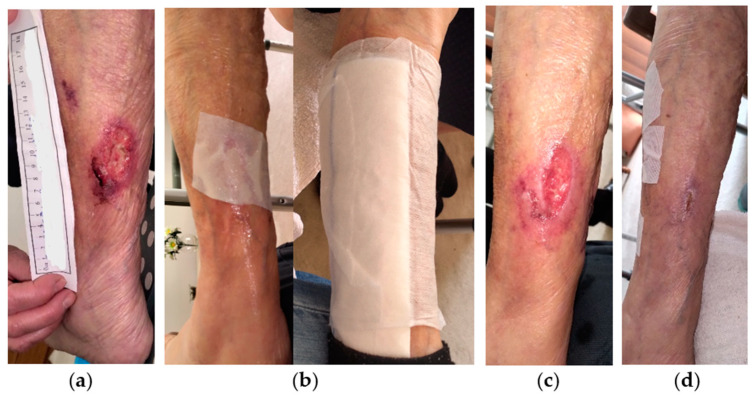
Case 5: (**a**) clinical findings at the initial examination, day 0 (start of MGH treatment); (**b**) wound dressing with L-MS, followed by L-MT, and a secondary foam dressing; (**c**) follow-up examination after 2 weeks, with the appearance of healthy granulation tissue and epithelialization; (**d**) complete wound healing after 6 weeks of MGH therapy.

An 87-year-old female patient presented with a stage II VLU on her left leg (Figure 5a). Medical history included CVI, AHT, peripheral artery disease (PAD), hyperthyroidism, hyperlipidemia, arthritis, and osteoporosis, while receiving anticoagulant (fondaparinux sodium 2.5 mg/0.5 mL) and glucocorticoid (methylprednisolone 4 mg) therapy. The VLU was in situ for 1 week without any wound care. On initial evaluation, the wound dimensions were 5 cm in length and 4 cm in width, and it presented with rolled, macerated edges associated with slough and low levels of exudate. Local dressing included L-MS, followed by L-MT and a secondary foam dressing, while changes were performed by the healthcare professional at the patient’s home twice a week (Figure 5b). Within 2 weeks, the strong healing properties provided by the MGH products allowed rapid cleansing of the wound bed and effective healing progress (Figure 5c). During the next days, the wound healing further progressed uneventfully, and the VLU healed completely after 6 weeks of MGH treatment (Figure 5d).

### 2.6. Case 6

**Figure 6:**
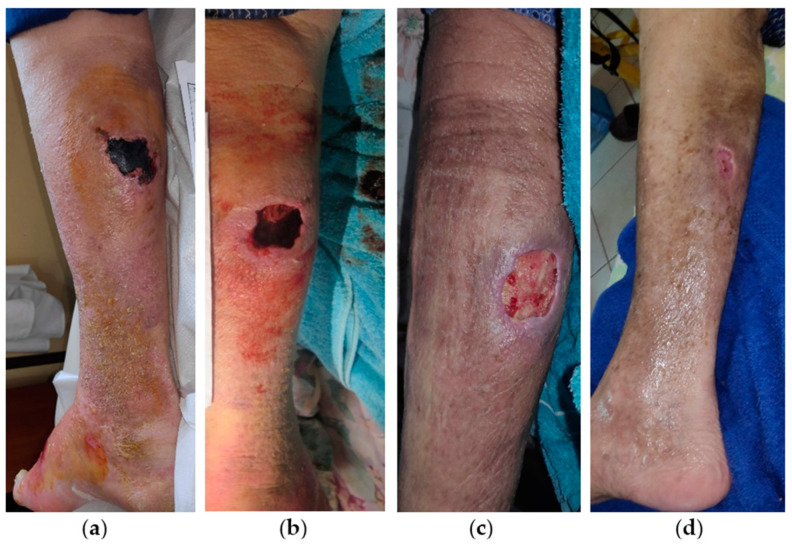
Case 6: (**a**) clinical findings at the initial examination, day 0 (start of MGH treatment); (**b**) wound bed after surgical debridement and drainage of the subcutaneous hematoma; (**c**) after 2 weeks of MGH therapy, the defect was already filled with healthy granulation tissue, without any sign of inflammation; (**d**) complete and uneventful wound healing after 11 weeks of MGH therapy.

An 83-year-old male patient presented with a large subcutaneous mass covered by a thick necrotic eschar at the posterior surface of his right leg (Figure 6a). Medical comorbidities included chronic CVI, AHT, heart failure, atrial fibrillation, and type 2 diabetes mellitus (T2DM), while he was under anticoagulant therapy (rivaroxaban 20 mg). The lesion had been present for 2 weeks without any local treatment. Initially, the wound dimensions were 5 cm in length and 5 cm in width, and surgical debridement of the necrotic tissue and drainage of the underlying hematoma was performed at the bedside (Figure 6b). Subsequently, the wound bed was meticulously lavaged with saline solution (NaCl 0.9%) and povidone-iodine solution. Given the large and deep skin and soft tissue defect, local treatment was started with L-MS in direct contact with the wound, followed by an alginate ribbon and a superabsorbent dressing pad to manage the secretions and prevent the recurrence of the bleeding. Wound dressing changes were performed by the healthcare professional at the patient’s home at 48 h intervals. After 2 weeks, exudate was considerably reduced, and healthy granulation tissue was evident and started to fill the defect (Figure 6c). Due to improved wound healing, wound dressings continued with L-MS, followed by L-MT and a secondary dressing pad, and performed every 4 days. The VLU was completely healed after 11 weeks of MHG treatment without complications (Figure 6d).

### 2.7. Case 7

**Figure 7:**
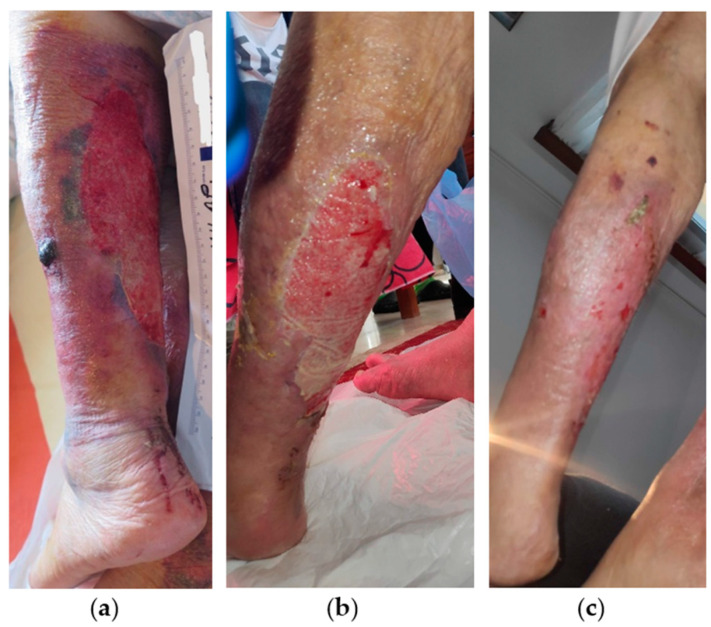
Case 7: (**a**) clinical findings at the initial examination, day 0 (start of MGH treatment); (**b**) reduction of the size of the wound defect and advanced wound healing after 2 weeks of MGH therapy; (**c**) complete and uneventful wound healing after 3 weeks of MGH therapy.

An 88-year-old female patient presented with a stage II VLU at the posterior surface of her left leg (Figure 7a). Medical comorbidities included chronic CVI, AHT, heart failure, atrial fibrillation, asthma, hyperlipidemia, and obesity. Moreover, the patient presented with permanent immobility and received anticoagulant (apixaban 2.5 mg) and glucocorticoid (methylprednisolone 4 mg) therapy. The VLU was in situ for more than 16 weeks, treated mainly with antiseptic solutions and antibiotic creams, and covered with gauze. On initial observation, the wound dimensions were 17 cm in length and 10 cm in width and presented with slough, moderate amount of exudate, edema, pain, and diffuse hematoma covering almost the entire surface of the leg. Treatment with L-MS, followed by L-MT and a superabsorbent dressing pad, was commenced, and dressing changes were performed at the patient’s home by the healthcare professional twice a week. After 2 weeks, the wound area reduced considerably in size, with resolution of the subcutaneous hematoma (Figure 7b). Dressing changes were continued as per the above protocol, and the VLU was completely healed uneventfully after 3 weeks (Figure 7c).

### 2.8. Case 8

**Figure 8:**
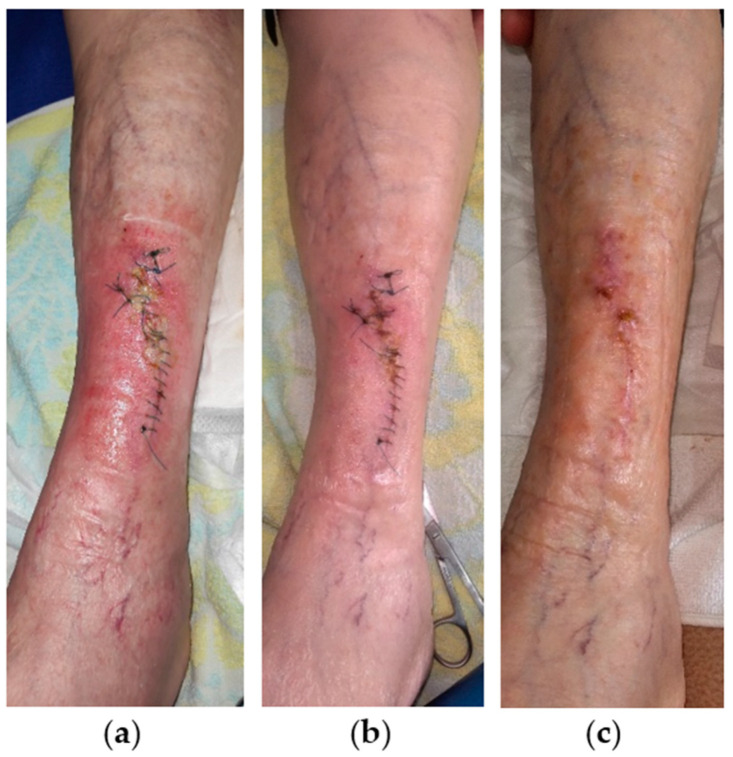
Case 8: (**a**) clinical findings at the initial examination, day 0 (start of MGH treatment); (**b**) resolution of the local infection, and advanced wound healing after 1 week of MGH treatment; (**c**) complete wound healing with the formation of a stable scar after 3 weeks of MGH treatment.

An 84-year-old female patient presented with a stage II VLU at the anterior surface of her left leg (Figure 8a). The patient’s medical history included CVI, postmenopausal osteoporosis, multiple myeloma, and hyperuricemia, while she was under anticoagulant (tinzaparin sodium 4500 antiXA iu/0.45 mL, acetylsalicylic acid 100 mg) and glucocorticoid (methylprednisolone 16 mg) therapy. Initially, the wound dimensions were 12 cm in length and 1 cm in width, and the wound was sutured. After 2 weeks of failed treatment with a povidone-iodine solution, the wound presented with dehiscence at its upper third and with signs of local infection, including slough, a moderate amount of exudate, and peripheral erythema. Local treatment was started with L-MS, followed by L-MT and a secondary foam dressing. Wound dressing changes were performed by the healthcare professional at the patient’s home at 48 h intervals. Already after 1 week, the infection resolved, and the wound area appeared healthy (Figure 8b). Consequently, dressing change intervals were extended to every 4 days. The healing progress was rapid and uneventful, and the VLU was completely healed after 3 weeks of MGH treatment with the formation of a stable scar (Figure 8c).

### 2.9. Case 9

**Figure 9:**
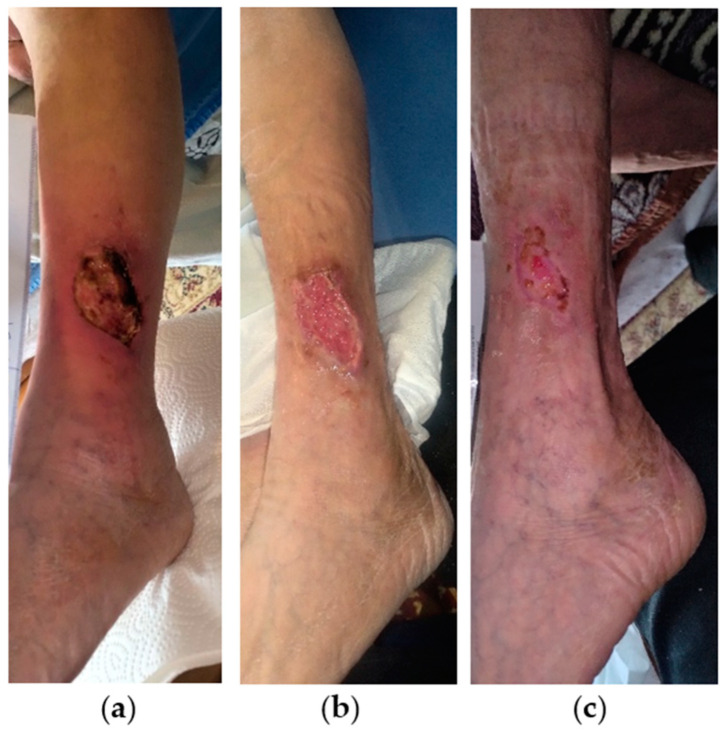
Case 9: (**a**) clinical findings at the initial examination, day 0 (start of MGH treatment); (**b**) resolution of the local infection, and rapid wound healing progress after 2 weeks of MGH therapy; (**c**) complete wound healing after 6 weeks of MGH treatment.

A 75-year-old female patient presented with a stage III VLU on the outer surface of the lower third of her left leg (Figure 9a). Medical comorbidities included CVI, AHT, T2DM, anemia, polymyalgia rheumatic, and depression. The patient suffered from limited mobility and was under chronic glucocorticoid therapy (methylprednisolone 4 mg). The VLU was treated locally with silver sulfadiazine for about 4 weeks, without any improvement. On initial observation, the wound dimensions were 6 cm in length and 5 cm in width and presented with signs of local infection including necrotic eschar, slough, moderate amount of exudate, and associated with peripheric signs of inflammation including erythema, edema, and pain. Treatment with L-MS, followed by L-MT, and a foam dressing was commenced, and dressing changes were performed every 2 days at the patient’s home by the healthcare professional. During the next 2 weeks, local signs of infection gradually disappeared, peripheric inflammation resolved, and the wound area was replaced by healthy granulation and epithelial tissue (Figure 9b). Due to the positive therapeutic response, dressing changes were extended to every 4 days. After 6 weeks, the ulcer was completely healed uneventfully (Figure 9c).

### 2.10. General Population Data

Table 1 shows an overview of the demographic data and wound characteristics. In total, nine patients (eight female and one male) with an average age of 83.4 years (range: 75–91 years) were included. All patients suffered from chronic venous insufficiency, while other common comorbidities included arterial hypertension and heart failure. All wounds showed local signs of infection, including slough, pain, and moderate to high amount of exudate. All VLUs treated with MGH showed resolution of the local infection within a mean time of 2.2 weeks (range: 1–4 weeks; median: 2 weeks) and were completely healed without any complication within a mean time of 7 weeks (range: 3–18 weeks; median: 6 weeks).

## 3. Discussion

This prospective case series included nine patients (eight female/one male) with an average age of 83.4 years (range: 75–91 years). All patients presented with multiple comorbidities (particularly cardiovascular diseases). A total of 11 VLUs were present, of which 7 wounds were partial thickness and 4 wounds were full thickness. MGH products were successfully used as a monotherapy in all patients. Clinical signs of infection were eliminated by MGH after 2.2 weeks on average (range: 1–4 weeks), and wounds were completely healed after 7 weeks on average (range: 3–18 weeks).

VLUs are typically located over the distal half of the lower leg. The wound bed is shallow with slough and exudate, while the surrounding skin presents with hemosiderin pigmentation, lipodermatosclerosis, and white atrophy [19]. Moreover, patients with CVI experience varicose veins, edema, pruritus, and burning pain in the leg [19]. These clinical findings are characteristics of the VLUs associated with CVI and constitute an important diagnostic tool for the evaluation of patients with leg ulcers. Therefore, the differential diagnosis during the evaluation of a patient with VLU must include conditions that can be complicated by a leg wound. The most common causes of lower leg ulcers are peripheral artery disease (PAD), metabolic disorders (mainly diabetic neuropathy), connective tissue diseases (such as pyoderma gangrenosum and rheumatoid arthritis), hematological diseases (such as chronic hemolytic anemias, and thalassemia), skin cancer (basal cell carcinoma, squamous cell carcinoma, Marjolin’s ulcer), infections (herpes, pyogenic, osteomyelitis, tuberculosis), prolonged pressure, and trauma [19,20,21].

In our study, major risk factors for the development of VLUs were female sex, advanced age, history of CVI, multiple comorbidities (mainly cardiovascular diseases), and limited mobility. Similar risk factors have been associated with VLUs and delayed wound healing [22,23,24]. Other risk factors for VLUs are higher body mass index (BMI > 25 kg/m2), history of superficial/deep venous thrombosis, number of pregnancies, physical inactivity, depression, and ulcer history [25,26,27]. Interestingly, all our patients received anticoagulant and/or glucocorticoid drugs to treat an underlying disease. One study identified anticoagulant use as a significantly independent risk factor for VLU infection, while it is well established that the chronic use of glucocorticoids can cause skin atrophy [28,29].

VLUs can be colonized by different microorganisms, delaying the wound healing process. The prevalence of infection in chronic wounds is up to 27% of the population, where tissue hypoxia and tissue necrosis are more pronounced [28]. The most common Gram-positive microorganism isolated in infected leg ulcers is *Staphylococcus aureus*, while *Pseudomonas aeruginosa* and *Escherichia coli* predominate among Gram-negative microorganisms [30]. All our patients presented with clinical signs of local infection or microbial colonization, while previous treatments with antiseptic solutions or antibiotic creams were used for a long time without any success. Using MGH products, we were able to gradually remove the necrotic tissue and slough, decrease the amount of exudate, eliminate the malodor, and reduce the pain from the wounds. At the same time, no adverse effects were noticed with the use of MGH. Consequently, we achieved complete resolution of the local infection in a mean time of 2.2 weeks (range: 1–4 weeks), in concordance with previous studies using the same MGH products [31,32]. Other studies showed a reduction in the ulcer size, pain, and malodor within 6–12 weeks, and higher satisfaction for patients treated with MGH compared with compression therapy as standard wound therapy [33,34]. MGH can inhibit bacterial colonization and therefore prevent clinical infection in hard-to-heal leg ulcers, without the need for systemic antibiotic administration.

The presence of biofilm in chronic wounds, which is a bacterial community surrounded by an extracellular matrix, can be up to 78.2%, whereas only 6% of acute wounds have biofilm [35]. MGH has a broad-spectrum antimicrobial activity and can effectively penetrate and destroy the barrier formed by different microorganisms, where antibiotics and antiseptics are ineffective [36]. Its high sugar content creates an osmotic gradient, inducing dehydration and growth inhibition of the bacteria [37]. Moreover, MGH stimulates the production of hydrogen peroxide and the release of molecules such as flavonoids, methylglyoxal, and bee defensin-1, leading to microorganism apoptosis with preservation of the surrounding healthy tissue [37]. Additionally, we used MGH products that are supplemented with vitamins C and E to further enhance the antimicrobial activity of raw honey. Given the multiple antimicrobial mechanisms of MGH, it is thought to be unlikely that microorganisms can develop resistance to MGH products.

In all presented cases, VLUs were healed completely and uneventfully, within a time ranging from 3 to 18 weeks (mean 7 weeks; median 6 weeks). Recently published case series have demonstrated similar healing times for VLUs treated with the same MGH products used in the current study [18,31]. Reduction of the wound size in the first 4 weeks, as well as complete ulcer healing within 12 weeks, should be the goal of the treatment strategy. The pro-healing effect of MGH is supported by its ability to induce osmosis, lower the wound pH, and its specific flavonoid content, leading to inhibition of leukocyte-produced matrix metalloproteinases (MMPs) that appear to be responsible for the degradation of the extracellular matrix (ECM) and cell-growth-promoting agents in chronic wounds [38,39]. Consequently, MGH can enhance wound healing outcomes, stimulating the formation of healthy granulation tissue, neo-vascularization, and re-epithelialization.

The MGH products used in our study were L-Mesitran^®^ Soft wound gel applied inside the wound and L-Mesitran Tulle^®^ to ensure contact with the wound bed and enhance the action of L-Mesitran Soft^®^. These MGH products are non-adherent, thereby avoiding trauma with removal, and comfortable to wear. Moreover, they are safe, with low costs, and simple to use, making the process of dressing changes easy for the patients and their relatives in a home care setting. MGH can be used as a monotherapy or in combination with other therapeutic approaches. Gethin et al. conducted a randomized clinical trial (RCT) showing better healing outcomes for VLU patients treated with MGH-impregnated compression bandaging over hydrogel dressings and compression bandaging at 12 weeks [40]. Nevertheless, Jull et al. also performed an RCT showing equivalent healing outcomes in VLU patients treated with MGH and compression bandaging when compared to calcium alginate dressings and compression bandaging [41]. Moreover, MGH products can be used for different indications in different age groups and in patients with already existing conditions, such as diabetics [42,43,44].

During the follow-up period of our study, no recurrence or complications related to VLUs were observed. While the average healing time of VLUs treated with different methods can be protracted, up to 6–12 months, different studies have reported recurrence rates ranging from 75% within 3 weeks to 70% within 5 years [2,5,45]. Despite the advanced age of our patients and the presence of multiple comorbidities, we were able to control local infection and offer an uneventful and rapid wound healing process, preventing the development of complications such as cellulitis, exposed tendons, osteomyelitis, and amputation.

## 4. Materials and Methods

### 4.1. Patient Recruitment

In this prospective observational case series study, we included nine patients (eight female and one male) with a total of 11 VLUs. Inclusion criteria were having a VLU not responding to previous therapies, the presence of local signs of bacterial contamination or infection, and patient consent. Exclusion criteria were having an allergy to bee stings or MGH, systemic signs of infection or inflammation, and patient non-consent.

The average age was 83.4 years (range: 75–91 years; median: 84 years), and all patients suffered from CVI and multiple comorbidities. Seven VLUs were partial-thickness and four were full-thickness defects. In eight patients, MGH products were used as a monotherapy, while in one patient, MGH products were used in combination with surgical (scalpel) debridement at the bedside. All patients were recruited prospectively over 59 months (February 2019 to January 2024), and the follow-up period was extended 4 weeks after complete wound healing was achieved, to notice any recurrence.

All wounds were treated at the patient’s home by a wound care professional following local wound care protocol. Wound characteristics and photographic documentation at the initial presentation and subsequent follow-up visits were collected and reviewed to assess the wound infection response to MGH therapy and evaluate the wound healing progress. Patient demographic data and wound course overview of all patients are summarized in Table 1. Data were analyzed in Excel by using formulas for the average and median.

### 4.2. L-Mesitran Wound Care Products

Triticum Exploitatie BV (Maastricht, the Netherlands) manufactures a range of MGH-based products designed to treat different types of skin wounds. L-Mesitran Soft (L-MS) is a hydro-active antibacterial wound gel containing 40% MGH (organic honey according to the MGH standards [12]), vitamins C and E, lanolin, propylene glycol, and PEG4000. L-MS is applied in direct contact with the wound, creating a moist wound-healing environment. L-Mesitran Tulle (L-MT) is a non-adhering antibacterial dressing impregnated with L-MS gel. L-MT enhances the effect of L-MS and prevents the secondary dressing from adhering to the wound bed. Both L-MS and L-MT facilitate autolysis of necrotic and devitalized material, providing bacterial growth inhibition and promoting the wound healing process. In all presented cases, L-MS and L-MT were applied in combination and covered with a secondary foam dressing to control the exudate and protect the peri-wound skin from maceration.

## 5. Conclusions

In our prospective case series, we successfully used MGH-based products to treat clinically infected VLUs in elderly patients with limited mobility and multiple comorbidities. VLUs are complex wounds with high morbidity, and the treatment approach must take into consideration either the modification of the risk factors or the appropriate local therapy. MGH was able to effectively resolve local infection and promote a rapid healing process. Moreover, MGH products are safe, cost-effective, and can be easily used by the patients and their relatives in a home care setting, improving the patient’s quality of life.

## Figures and Tables

**Table 1:** Patients’ demographic data and wound characteristics.

Case	Gender/Age (Years)	VLU Location/Dimensions (cm)	Wound Age (Weeks)/ Previous Treatment	Relevant Comorbidities	Local Signs of Infection	Time for Infection Resolution (Weeks)	Time for Wound Healing (Weeks)
1	Female 79	Right 6 × 6	4 povidone-iodine solution and mupirocin calcium cream	CVI, AHT, COPD, heart failure, atrial fibrillation, obesity, limited mobility	slough, high amount of exudate malodor, pain, erythema, delayed healing	4	11
2	Female 80	Right 3 × 3	20 povidone-iodine solution	CVI, AHT, obesity	slough, moderate amount of exudate, edema, delayed healing	3	18
3	Female 91	Right: 5 × 4 Left lateral: 3 × 3 Left posterior: 6 × 5	2 povidone-iodine solution	CVI, DVT, heart failure, atrial fibrillation, limited mobility	necrotic tissue, moderate amount of exudate, erythema, edema, pain	Right: 3 Left posterior: 2 Left lateral: 1	Right: 5 Left posterior: 6 Left lateral: 3
4	Female 84	Right 9 × 6	4 not treated	CVI, COPD, CVD, heart failure, atrial fibrillation, obesity, permanent immobility	necrotic tissue, malodor, edema, pain moderate amount of exudate, erythema, hyperthermia, delayed healing	2	4
5	Female 87	Left 5 × 4	1 not treated	CVI, AHT, PAD, limited mobility	slough, low amount of exudate, rolled and macerated edges	2	6
6	Male 83	Right 5 × 5	2 not treated	CVI, AHT, T2DM, heart failure, atrial fibrillation, limited mobility	necrotic tissue, edema, erythema, pain	2	11
7	Female 88	Left 17 × 10	>16 antiseptic solutions & antibiotic creams	CVI, AHT, heart failure, atrial fibrillation, obesity, permanent immobility	slough, moderate amount of exudate, edema, pain, delayed healing	2	3
8	Female 84	Left 12 × 1	2 povidone-iodine solution	CVI, multiple myeloma, limited mobility	slough, erythema, moderate amount of exudate	1	3
9	Female 75	Left 6 × 5	4 silver sulfadiazine cream	CVI, AHT, T2DM, limited mobility	necrotic eschar, slough, moderate amount of exudate, erythema, edema, pain, delayed healing	2	6

VLU: venous leg ulcer, CVI: chronic venous insufficiency, AHT: arterial hypertension, COPD: chronic obstructive pulmonary disease, DVT: deep vein thrombosis, CVD: cerebrovascular disease, PAD: peripheral artery disease, T2DM: type 2 diabetes mellitus.

## Data Availability

The data that support the findings of this study are available from the corresponding author upon reasonable request. All data relevant to the study are included in the article.
